# Acute Hematological Toxicity during Cranio-Spinal Proton Therapy in Pediatric Brain Embryonal Tumors

**DOI:** 10.3390/cancers14071653

**Published:** 2022-03-24

**Authors:** Sabina Vennarini, Giada Del Baldo, Stefano Lorentini, Riccardo Pertile, Francesco Fabozzi, Pietro Merli, Giacomina Megaro, Daniele Scartoni, Andrea Carai, Assunta Tornesello, Giovanna Stefania Colafati, Antonella Cacchione, Angela Mastronuzzi

**Affiliations:** 1 Proton Therapy Center, Hospital of Trento, Azienda Provinciale per I Servizi Sanitari (APSS), 38123 Trento, Italy; sabina.vennarini@istitutotumori.mi.it (S.V.); stefano.lorentini@apss.tn.it (S.L.); daniele.scartoni@apss.tn.it (D.S.); 2 Pediatric Radiotherapy Unit, Fondazione IRCCS Istituto Nazionale dei Tumori, 20133 Milan, Italy; 3 Department of Paediatric Haematology/Oncology, and Cell and Gene Therapy, Bambino Gesù Children’s Hospital, IRCCS, 00165 Rome, Italy; giada.delbaldo@opbg.net (G.D.B.); francesco.fabozzi@opbg.net (F.F.); pietro.merli@opbg.net (P.M.); giacomina.megaro@opbg.net (G.M.); antonella.cacchione@opbg.net (A.C.); 4 Department of Clinical and Evaluative Epidemiology-Trento Health Service, Azienda Provinciale per I Servizi Sanitari (APSS), 38123 Trento, Italy; riccardo.pertile@apss.tn.it; 5 Neurosurgery Unit, Department of Neurosciences, Bambino Gesù Children’s Hospital (IRCCS), 00165 Rome, Italy; andrea.carai@opbg.net; 6 Pediatric Oncology Unit, Ospedale Vito Fazzi, 73100 Lecce, Italy; oep.polecce@ausl.le.it; 7 Neuroradiology Unit, Department of Imaging, Bambino Gesù Children’s Hospital (IRCCS), 00146 Rome, Italy; gstefania.colafati@opbg.net

**Keywords:** proton therapy, embryonal tumors, craniospinal irradiation, acute hematological toxicity, childhood brain tumors

## Abstract

**Simple Summary:**

Embryonal tumors include a heterogeneous group of tumors that need multimodal and multidisciplinary treatments in which craniospinal irradiation (CSI) plays a major role, with a known impact on the acute toxicity and future quality of life of patients. Neutropenia represents one of the most common acute hematological side effects and is responsible for infections and treatment delays that can affect the effectiveness of therapy. To better describe hematological acute toxicity during proton beam radiation treatment, we retrospectively examined 20 subsequent pediatric patients affected by high-risk embryonal tumors subjected to CSI with dual-phase proton therapy after a chemotherapy regimen. Our data suggest that the dual-phase technique is safe and feasible in this setting of pediatric patients with a significant baseline hematological toxicity. Despite all patients having undergone chemotherapy prior to irradiation, no serious hematological toxicity was reported at the end of the treatment with proton therapy, and, therefore, no treatment was discontinued or delayed.

**Abstract:**

Background: Embryonal tumors represent a heterogeneous entity of brain tumors that need a multidisciplinary treatment including cranio-spinal irradiation (CSI), with a known impact on the acute toxicity. Proton therapy (PT) boasts a reduction in acute hematological toxicity. Methods: We retrospectively examined 20 pediatric patients affected by high-risk medulloblastoma and other rare embryonal brain tumors subjected to CSI with PT from September 2016 to April 2020. Before CSI, all patients received induction chemotherapy, and three patients additionally received two high-dose courses with thiotepa, followed by an autologous haemopoietic stem cell transplantation. We recorded the total white blood cell count, absolute neutrophil count, platelets, and hemoglobin levels for all patients during PT. Results: Leucocytes and neutrophils decreased directly after the beginning of treatment, reaching a complete recovery at the end of treatment. Hemoglobin values remained constant over the treatment course. The median platelet value decreased until reaching a plateau around halfway through therapy, followed by a slow increase. No cases of febrile neutropenia or severe infections were reported. No treatment discontinuation due to hematological toxicity was necessary. Conclusions: CSI with PT was proven to be safe in this setting of pediatric patients. Our study showed that despite all patients having undergone chemotherapy prior to irradiation, no serious hematological toxicity was reported at the end of the treatment with PT, and, therefore, no treatment was discontinued or delayed.

## 1. Introduction

Central nervous system (CNS) tumors represent 15–20% of all malignant neoplasms in children; they are the second leading cause of childhood cancer and are the most frequent solid tumors in the pediatric age as well as the leading cause of cancer-related morbidity and mortality [1].

Embryonal tumors, including medulloblastoma (MBs), account for 20% to 25% of pediatric CNS tumors [2]. In the last few years, methylation assays and molecular profiling have shown a large spectrum of heterogeneous entities that led to their recent reclassification [3], and the term CNS primitive neuroectodermal tumor (CNS-PNET) was removed from the 2016 World Health Organization (WHO) classification of CNS tumors [4]. Embryonal tumor treatment is still challenging and requires a multidisciplinary approach, including surgery, chemotherapy, and radiotherapy [5,6,7]. Survival rates with the current standard of care are approximately 50–75% for high-risk MBs [8] and 30–50% for other embryonal tumors [9].

Cranio-spinal irradiation (CSI) is recommended for tumors with the tendency to seed the subarachnoid spaces of the brain, spine, and cerebrospinal fluid, as this is considered a potential reservoir of neoplastic cells, and those with documented metastatic dissemination [10]. Although the efficacy of CSI has been well recognized in some entities among brain tumors, this treatment is not without risks. Acute, subacute, and long-term neurologic, endocrine, and cognitive complications can often occur.

Compared with traditional photon radiotherapy, proton therapy (PT) can reduce the volume irradiated and, consequently, the radiation dose to normal tissue, with a decreased risk of acute and long-term toxic effects but with the same level of disease control [11].

Concerning long term toxicity, it is well known that CSI PT is less toxic in comparison to photon irradiation. Severe hearing loss is significantly lower in MB patients treated by PT rather than radiotherapy. The mean average intelligence quotient score is described to be lower in the PT series than in several different photon-treated cohorts. Moreover, hormonal deficits are less frequent than in MB treated with photon therapy. Cardiac, pulmonary, or gastrointestinal late toxic effects are also extremely rare [11].

In the context of acute toxicity during CSI, the study of hematological toxicity has always been of great interest [12]; however, little is known about how this may impact survivors’ lives.

Moreover, in the main therapeutic protocols in use, CSI is generally preceded by intense chemotherapy schemas that lead to a severe depletion of vital hematological reserves. Acute radio-induced damage manifests clinically as cytopenias and depends on several factors, such as the irradiated volume, delivered radiation dose, dose rate, and interaction with chemotherapy [12,13,14,15,16]. Considering that the craniospinal axis is contained in more than half of the hematopoietically active bone marrow, it becomes self-evident that hematologic toxicity is one of the main adverse effects of CSI [13]. Particularly, neutropenia represents one of the most common side effects and is responsible for both infections and treatment delays [13,14,17].

Compared to CSI irradiation with photons, the CSI PT technique offers a considerable sparing of the hematopoietic bone marrow dose volumes in children of any age who are candidates for this radiation approach [7]. These physical advantages determined by the characteristics of proton particles could clinically translate into better tolerability of CSI and a lower depletion of the hematopoietic reserves during treatment, reducing the risk of neutropenia and infections [15,18,19]. 

The purpose of the work is to describe an observational retrospective study on the acute toxicological trend of the main peripheral circulating hematological lines during a curative dose of proton beam irradiation of the craniospinal axis in children with different types of embryonal tumors.

## 2. Materials and Methods

### 2.1. Patient Selection

We retrospectively examined a mono-institutional study of pediatric patients affected by high-risk MB and other rare embryonal brain tumors (pineoblastomas and CNS neuroblastoma) diagnosed at Bambino Gesù Children’s Hospital and subjected to CSI with PT at the Center of Proton Therapy in Trento (Italy).

High-risk MB was defined as patients who had a subtotal resection (>1.5 cm^2^), and/or evidence of metastatic disease, and/or unfavorable histology (anaplastic/large cell morphology), and/or MYC-MYCN amplification status.

The extracted patient data included: age, sex, histology, surgical details, disease staging (localized or metastatic), MYC status, chemotherapy and proton treatment details, and blood count data at different timepoints.

This study was approved by the institutional review boards of the Bambino Gesù Children’s Hospital. Written informed consent was obtained from all the patients or legal guardians.

### 2.2. Surgery and Chemotherapy

All patients were subjected, after surgery or biopsy, to the same chemotherapy regimen, including methotrexate, vincristine, cyclophosphamide, etoposide, and carboplatin, in accordance with the national recommendation [7,20], and in selected cases, two courses of high-dose chemotherapy with thiotepa were followed by an autologous hematopoietic stem cell transplantation (HSCT). 

### 2.3. Radiation Treatment Planning

**Figure 1:**
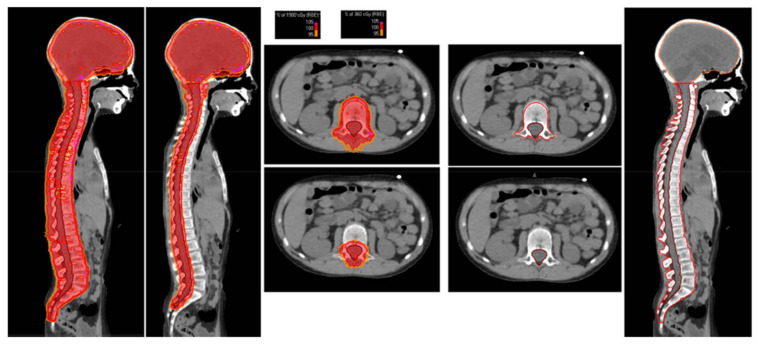
The right side shows the target volumes drawn for the contouring of the CSI. The left side reports the dose distributions of the first phase (19.8 GyRBE to the brain plus the whole vertebral body) and the second phase (16.2 GyRBE to the brain plus the subarachnoid space and the spinal nerve roots) of the CSI.

Patients were treated in a supine position using thermoplastic masks coupled with customized neck support and knee fixation support to guarantee immobilization during treatment. For all patients who had not completed skeletal maturity, namely patients ≤15 years of age (19 out of 20), in order to prevent uneven growth, a uniform dose to the entire vertebrae was administered; the clinical target volume (CTV) was represented by the whole brain, including the cribriform plate and the first third of the optic nerves as well as the entire spine, including the subarachnoid space, vertebral body, and spinal nerve roots; inferiorly, the edge of the CTV was set at the end of the thecal sac (usually at S2-S3 level). Our approach to CSI involved two steps of the dose (dual-phase) for the spinal region irradiation (Figure 1). 

All patients received a total CSI dose of 36 GyRBE. Those who had not reached skeletal maturity received the first 19.8 GyRBE (i.e., the first 11 treatment fractions of the CSI) to the entire vertebral body, while the remaining 16.2 GyRBE (i.e., the last 9 fractions of the CSI) were delivered only to the subarachnoid space and the spinal nerve roots. The planning target volume (PTV) was defined as a CTV expansion of 3 mm in the anterior-posterior direction and 5 mm in the latero-lateral and inferior-superior directions for the first treatment phase (up to 19.8 GyRBE), while for the second phase (the remaining 16.2 GyRBE) PTV was defined as a 5 mm isotropic expansion of the CTV.

Concerning treatment planning, the arrangement of the fields was calculated with two lateral plus a posterior beam for the brain irradiation, together with a posterior beam (actually one or two, depending on target length in the inferior-superior direction) to cover the spine region. A single-field optimization technique was applied to ensure robust target coverage. Details of the planning technique that was used have been published in the past and are available in the literature [21]. 

The proton facility is equipped with a 226 MeV cyclotron (Iba Proteus Plus) featuring a pencil beam scanning delivery technique and 360° gantry machines [22].

### 2.4. Hematological Data

The following hematological tests were performed: total white blood cell count, absolute neutrophil count, platelets, and hemoglobin levels. These data were collected at the baseline and endpoint of the CSI-PT treatment and at least once per week from each patient during radiation treatment. The hematological data are given in the Supplementary Materials.

Leukopenia, neutropenia, anemia, and thrombocytopenia were classified according to the National Cancer Institute’s Common Terminology Criteria for Adverse Events (CTCAE) v5.0 [23].

### 2.5. Data Analysis and Statistics

A descriptive analysis was carried out, representing the quantitative variables in terms of the mean values together with their confidence intervals at 95%, standard deviation, and range (minimum-maximum) for each time point relative to the following treatment fractions: baseline, 3rd–7th fractions, 8th–11th fractions, 12th–15th fractions, 16th–20th fractions, 21st–27th fractions, and the end of treatment (endpoint). The values of leucocytes, hemoglobin, platelets, and neutrophils over the treatment course were then graphically displayed. The comparisons of the median values of the hematological parameters scored at the beginning and the midpoint (from the 8th to 11th fractions) of PT administration, the midpoint versus the end of therapy, and the beginning versus the end of treatment were performed using the Wilcoxon signed-rank sum test. The level of statistical significance was set to *p*-value ≤ 0.05. The software used for the statistical analysis was the SAS System version 9.4 (SAS Institute Inc., Cary, NC, USA). 

## 3. Results

### 3.1. Patient Characteristics

Between September 2016 to April 2020, we enrolled 20 patients affected by high-risk MB (17/20), localized CNS neuroblastoma (1/20), and metastatic pineoblastoma (2/20) who were treated at the Bambino Gesù Children’s Hospital and subjected to CSI with PT at the Center of Proton Therapy in Trento (Italy). 

The median age at diagnosis was 6.95 years (range 3.2–18.43 year), and 13/20 (65%) patients were male. 

The metastatic stage of disease was reported in half of the population. The population are given in the Supplementary Materials.

### 3.2. Surgery and Chemotherapy

A surgical resection was performed on 18 patients at diagnosis (70% complete resection and 20% partial removal); biopsy was performed on 2 patients (10%) with metastatic pineoblastomas at onset.

All patients received standard chemotherapy after surgery according to the national recommendation [7,20]. In three cases, high-dose chemotherapy with thiotepa followed by autologous HSCT was administered to further reduce the disease burden before PT. 

### 3.3. Proton Therapy

All patients included in the study received CSI-PT at the total dose of 36 Gy RBE (delivered in 20 treatment fractions of 1.8 Gy RBE per fraction) at a median time after the end of chemotherapy of 31.5 days (IQR 27–36). Sequentially with CSI, patients received a boost on the primary site of disease at the dose of 18 Gy RBE (delivered in 10 treatment fractions of 1.8 Gy RBE per fraction) for a total dose of 54 Gy RBE. 

Three patients received a boost on the metastatic residues present at the pre-proton therapy imaging, concurrent with the boost on the primary site, at the overall dose of 45 GyRBE on the thoracic spine tract and 50.4 Gy RBE on the lumbosacral tract. Among these, one also received an 18 Gy RBE boost for a brain metastasis (Table 1). 

A summary of the clinical characteristics and treatments performed on the patients is shown in Table 1.

### 3.4. Blood Count

The mean (along with the standard deviation and 95% confidence interval), median, and maximum-minimum values for leukopenia, neutropenia, anemia, and thrombocytopenia were stratified for each selected timepoint. A comprehensive summary of the statistical analysis results performed on the data of the 20 patients enrolled in the study is provided in Table 2.

**Figure 2:**
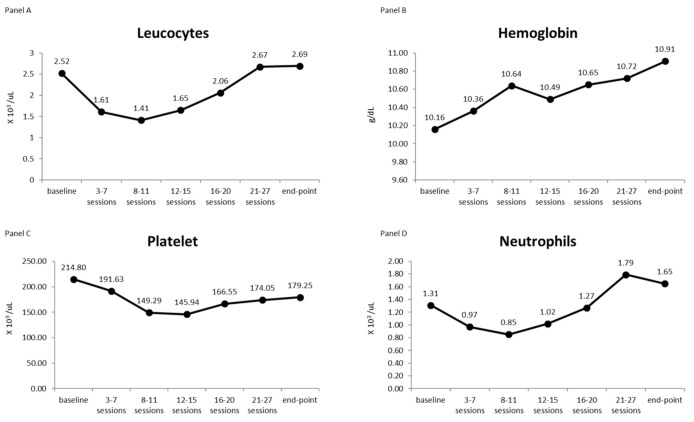
The four panels show the trend over time of the median values of leucocytes (panel **A**), hemoglobin (panel **B**), platelets (panel **C**), and neutrophils (panel **D**) as a function of the treatment sessions.

The variation of leucocytes, hemoglobin, platelets, and neutrophils over the treatment course in terms of median values is described in Figure 2. Panel A shows that leucocytes decreased directly after the beginning of treatment, reaching the minimum values around the 8th-11th treatment sessions, which corresponds to the conclusion of the entire vertebral body irradiation. Then, a continuous rise in leucocytes was observed, up to a complete recovery that made the values comparable to those scored at the beginning of the PT. As shown in Table 3, both the trends, i.e., the initial decrease until the midpoint of treatment and the final recovery between the midpoint and the end of the treatment, are statistically significant. Panel B shows that hemoglobin values remained constant over the treatment course, without any statistically significant variation, except when comparing the initial and final values, as reported in Table 3. Panel C describes the trend of the platelets over time: the median value decreased until reaching a sort of plateau around the 12–15th treatment sessions, followed by a slow increase. Table 3 reports that the difference between the median value of platelets at the beginning and at the midpoint of treatment as well as the difference between the values at the midpoint and at the end of PT were statistically significant. Finally, panel D shows how the neutrophils moved over time: initially they decreased, similarly to what happened to the leucocytes, reaching the minimum value at the end of the irradiation of the entire vertebral body (around 8–11th treatment sessions). Then, when the irradiation was restricted to the spinal channel only, a constant increase in the neutrophils was observed, reaching the end of the PT with values higher than those scored at the beginning of the therapy. Table 3 reports the statistical significance of the median value differences scored at the different considered time points.

**Figure 3:**
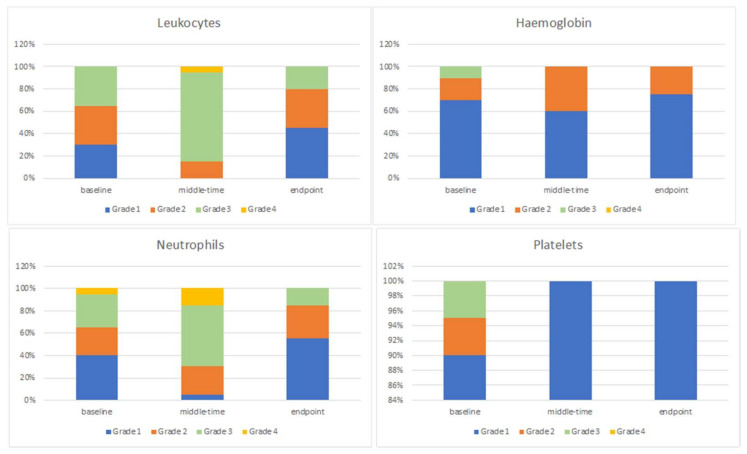
Grade of toxicity at different timepoints, classified according to CTCAE 5.0v for leukocyte, neutrophils, hemoglobin, and platelets.

As for the CTCA 5.0 classification, the percentages of patients with toxicity grades of 1, 2, 3, and 4 for each of the cell lines are summarized in Figure 3.

Febrile neutropenia and serious infectious complications were never reported. All patients underwent prophylactic treatment with acyclovir and trimethoprim sulfamethoxazole during PT.

Hospitalization was never necessary for any patient, and all patients underwent PT as an outpatient procedure. No patient was treated with supportive therapies during treatment, such as hematopoietic growth factors and/or blood transfusions or their derivatives, including as a precaution on the weekends.

Treatment radiation was never interrupted, ensuring an average irradiation timing of 42.75 days and a median of 42 days (range 41–48 days).

The acute toxicity detected in our series did not impact the overall well-being of patients, and the therapeutic decision by the entire clinical group was to perform a very close monitoring protocol according to the hematological data of the toxicity progressively detected in the course of our study.

## 4. Discussion

Embryonal tumors are a collection of biologically heterogeneous lesions that share the tendency to disseminate throughout the central nervous system via CSF pathways. For this reason, CSI radiation is a cornerstone of treatment together with surgery and chemotherapy, but its early and late sequelae are still a challenging matter. Developments in modern proton beam therapy are improving normal tissue dose-sparing while maintaining satisfactory target coverage, reducing the risk of side effects. 

Even low doses of radiation can easily damage the hematopoietic system. There are several mechanisms that can be attributed to the hematological toxicity: direct damage to hematopoietic stem cells and pathological changes in ancillary cells that regulate the hematopoietic process through the secretion of cytokines and growth factors, which are located in the stroma and the bone marrow microenvironment [16,24]. Radio-induced acute cytopenia is influenced by numerous factors (irradiated volume, delivered radiation dose, and dose rate) and previous chemotherapy. The timing of cytopenia onset differs between cell lineages, occurring first in lymphocytes, then neutrophils and platelets, and finally red blood cells [12,13,14,15,16]. Several factors contribute to these differences among blood cell populations, such as intrinsic radiosensitivity, the life span of peripheral cells, and the time required for their replacement [14,16]. 

Neutropenia represents one of the most common side effects, responsible for infection and treatment delays that can affect the effectiveness of therapy [13,17,25]. The use of hematopoietic growth factors (HGFs) can potentially help overcome this toxicity, as there is well-established evidence of their efficacy in preventing both febrile neutropenia and treatment delays in the chemotherapy-induced neutropenia setting. Furthermore, it should be emphasized that the use of HGFs should be avoided in patients receiving RT and concomitant chemotherapy because of the increased risk of complications and death [25,26]. Nevertheless, numerous issues regarding the actual efficacy and safety remain unanswered. It must be remembered that there are no specific contraindications to RT administration in patients with asymptomatic or afebrile neutropenia, while it is generally discontinued if febrile neutropenia occurs; however, specific data in this regard are limited [14]. 

To better describe hematological acute toxicity during proton beam radiation treatment, we retrospectively examined 20 subsequent pediatric patients affected by high-risk embryonal tumors subjected to CSI with dual-phase PT after a chemotherapy regimen. During hematological monitoring, we found that leucocytes and neutrophils decreased directly after the beginning of treatment, reaching the minimum values around the 8–11th treatment sessions, up to a complete recovery at the end of treatment. Hemoglobin values remained constant over time during treatment, and platelet values decreased until reaching a plateau around the 12–15th treatment sessions, followed by a slow recovery. 

According to the CTC AE 5.0v classification [23], the maximal grade of toxicity registered for leucocyte and neutrophils was grade 4 halfway through therapy in 5% and 15% of patients, respectively. The maximal grade of hemoglobin toxicity was grade 2 at the midpoint in 40% of patients. In all cases, the platelet counts were classified as grade 1 toxicity, both at the midpoint and at the end of treatment. Chang et al. reported treatment interruption with photon therapy in about 36% of their population. Grade 3–4 leukopenia was described in 76% of patients who received chemotherapy before radiotherapy, neutropenia in 50%, thrombocytopenia in 90%, and anemia in 24% [13]. In our cohort, a maximal grade 3–4 leukopenia was found in 35% of patients, and neutropenia was found in 70% at the midpoint of the treatment, with complete recovery at the end of treatment. Grade 3–4 thrombocytopenia and anemia were never recorded during treatment. A significative reduction in acute hematological toxicity with PT was already described [27]. 

In our cohort, no patient was administered HGFs or delayed treatment for infectious episodes, with a spontaneous recovery of the neutrophil count starting about halfway through the course. None of our patients required antibiotic therapy and/or hospitalizations for febrile neutropenia, compared to 4.5% reported in other studies [12].

Of note, none of the patients in our cohort even needed platelet or red blood cell transfusions. On the contrary, in a case series of adult and pediatric patients with different primary brain tumors treated with photon CSI, blood transfusions were necessary in about 10% of population during radiation treatment [12]. 

In our cohort, treatment radiation was never interrupted. Jefferies et al. described a photon radiotherapy interruption in 24.5% of the population (90% of cases due to leukopenia and 10% due to thrombocytopenia) [12], and Mac Manus reported treatment discontinuation for thrombocytopenia in a quarter of patients [28].

As expected, blood count depletion is more frequent in patients treated with chemotherapy before radiation treatment [12,13]. According to this sentence, Kumar et al. did not find grade 4 hematological toxicity, and grade 3 was described in 19% of patients affected by MB that did not receive chemotherapy before radiation; in fact, treatment discontinuation was observed in only 1.9% of patients (in 92% of cases it was caused by leukopenia) [14]. It is fair to point out that all our patients had received chemotherapy prior to the start of PT, and, in three cases, high-dose chemotherapy had been administered. Moreover, three patients needed a boost of irradiation for metastatic lesions, receiving a higher radiation load. Despite this, all patients improved their hematological counts at the end of proton radiation treatment, and radiation treatment was never discontinued. 

It is important to highlight that our approach to CSI involved two steps of dose (dual-phase) for what concerns the spinal region irradiation. This dual-phase approach aimed to deliver a homogeneous dose to the vertebral body, limiting it to a level (around 20 GyRBE), which allows for a uniform growth of the skeletal structure [21]. This vertebral body irradiation technique complied with the rules of good clinical practice to respect the growth of the child and avoid long-term sequelae before the completion of bone maturation [29]. Administering a homogeneous dose and delineating the ossification nuclei not only preserves the growth of children [30] but, at the same time, limits the dose to the vertebrae so that it can also be useful for safeguarding the child’s hematopoietic heritage and reducing hematological toxicity, as the vertebral body includes a considerable amount of bone marrow [17,31].

It was interesting in our study to see how from the 8^th^ to 11th sessions, which correspond to a dose range of 14.4–19.8 Gy (RBE), all the hematological values studied, except the hemoglobin value (whose rationale is due to the half-life of the red blood cell), had a significant decrease with respect to the base-line value and then slowly increased again with the completion of the craniospinal irradiation at the dose of 16.2 Gy (RBE), for a total of 36 Gy (RBE).

Our data suggest that the dual-phase technique, which allows for the administration of radiation treatment with few hematological toxicities, is safe and feasible.

Therefore, the novelty of the study, thanks to the detailed analysis of the trend of the hematological parameters, was to dictate a conservative behavior that did not deem it necessary to administer growth factors due to their potential long-term disadvantages, including the mobilization of the hematological stem cell reserve, which, if subsequently irradiated, would lead to a depletion of the cancer survivor’s future bone marrow reserve.

To our knowledge, this is one of the few studies detailing the hematological trends during craniospinal PT [11,15,18,19] in a population of pediatric patients with high-risk MB and other rare embryonal tumors.

Although the population is small, the study showed that, despite all patients having undergone chemotherapy prior to irradiation, no serious hematological toxicity was reported at the end of the treatment with dual-phase PT, and, therefore, no treatment was discontinued.

## 5. Conclusions

In our study, we demonstrated that the proton technique was proven to be safe in CSI, even in brain cancer patients with significant baseline hematologic toxicity due to previous chemotherapy. Moreover, patients did not require supportive therapy with transfusions or HGFs, and there were no delays in treatment. Finally, it saves patients additional therapies and time spent as inpatients, which can also be very important in improving the patient’s quality of life during medical care.

## Figures and Tables

**Table 1:** Patients’ characteristics and treatment details.

Age at Diagnosis	Median	Range
	6.95 years	3.2–18.43 years
	*n*	%
Sex		
Male	13	65
Female	7	35
Histology		
Medulloblastoma	17	85
Pineoblastoma	2	10
CNS Neuroblastoma	1	5
Metastatic disease		
Yes	10	50
No	10	50
Surgery		
Total resection	14	70
Subtotal resection	4	20
Biopsy	2	10
Chemotherapy		
Standard dose	20	100
High-dose chemotherapy and HSCT	3	15
Doses, Gy-RBE		
Craniospinal dose (19.8 + 16.2)	36	36 (all patients)
Total dose (CSI + boost primary site)	54	54 (all patients)
Boost Metastasis site (only 3 patients)	45	18–50.4

**Table 2:** The table reports the values of some statistical indexes (i.e., mean, standard deviation, minimum, maximum, and median) for leucocytes, hemoglobin, platelets, and neutrophils at different times over the proton treatment course. In between the baseline and end of treatment, the values of the parameters are provided as average values grouping several treatment sessions.

	Time	Mean	SD	95% C.I. for Mean		Min	Max	Median
Leucocytes	Baseline	2.52	0.97	2.07	2.97	1.40	5.10	2.35
	3rd–7th sessions	1.61	0.41	1.41	1.80	0.80	2.70	1.50
	8th–11th sessions	1.41	0.44	1.18	1.64	0.70	2.70	1.40
	12th–15th sessions	1.65	0.61	1.35	1.95	0.60	2.60	1.65
	16th–20th sessions	2.06	0.51	1.82	2.30	1.00	3.00	2.05
	21st–27th sessions	2.67	0.95	2.23	3.11	1.50	5.50	2.35
	Endpoint	2.69	0.91	2.26	3.11	1.10	4.50	2.50
Hemoglobin	Baseline	10.16	1.07	9.66	10.66	7.80	12.10	10.35
	3rd–7th sessions	10.36	0.78	9.98	10.74	9.00	12.30	10.40
	8th–11th sessions	10.64	0.67	10.29	10.98	9.20	12.00	10.70
	12th–15th sessions	10.49	0.86	10.06	10.92	8.60	11.70	10.70
	16th–20th sessions	10.65	1.25	10.06	11.23	8.00	13.20	10.65
	21st–27th sessions	10.72	1.01	10.25	11.19	8.90	12.80	10.65
	Endpoint	10.91	1.09	10.40	11.41	8.90	13.30	11.15
Platelets	Baseline	214.80	106.73	164.85	264.75	36.00	409.00	231.5
	3rd–7th sessions	191.63	78.12	153.98	229.28	74.00	370.00	184.0
	8th–11th sessions	149.29	40.47	128.49	170.10	74.00	241.00	150.0
	12th–15th sessions	145.94	49.76	121.20	170.69	79.00	264.00	140.0
	16th–20th sessions	166.55	55.76	140.45	192.65	86.00	272.00	164.5
	21st–27th sessions	174.05	57.51	147.14	200.96	70.00	290.00	161.0
	Endpoint	179.25	53.32	154.29	204.21	78.00	304.00	166.5
Neutrophils	Baseline	1.31	0.66	1.00	1.62	0.30	2.60	1.15
	3rd–7th sessions	0.97	0.35	0.80	1.14	0.40	1.90	1.00
	8th–11th sessions	0.85	0.36	0.66	1.03	0.30	1.90	0.80
	12th–15th sessions	1.02	0.52	0.76	1.27	0.10	1.80	1.00
	16th–20th sessions	1.27	0.49	1.03	1.50	0.10	2.30	1.25
	21st–27th sessions	1.79	0.74	1.44	2.13	0.90	3.80	1.60
	Endpoint	1.65	0.60	1.36	1.94	0.70	2.80	1.60

**Table 3:** Results of the statistical analysis performed with Wilcoxon signed rank sum test on the median values of leucocytes, hemoglobin, platelets, and neutrophils at baseline, the midpoint, and at end of treatment.

	Time	*p*-Value of Wilcoxon Signed-Rank Sum Test
Leucocytes	Baseline vs. Midpoint	<0.0001
	Midpoint vs. Endpoint	<0.0001
	Baseline vs. Endpoint	0.6408
Hemoglobin	Baseline vs. Midpoint	0.1896
	Midpoint vs. Endpoint	0.3958
	Baseline vs. Endpoint	0.0100
Platelets	Baseline vs. Midpoint	0.0046
	Midpoint vs. Endpoint	0.0008
	Baseline vs. Endpoint	0.0954
Neutrophils	Baseline vs. Midpoint	0.0005
	Midpoint vs. Endpoint	<0.0001
	Baseline vs. Endpoint	0.2502

## Data Availability

The data presented in this study are available in the Supplementary Material.

## Supplementary Materials

The following supporting information can be downloaded at: https://www.mdpi.com/article/10.3390/cancers14071653/s1, Supplementary Materials: hematological data and population Clinical data.
